# Characterization of emerging H3N3 avian influenza viruses in poultry in China

**DOI:** 10.1080/22221751.2025.2509748

**Published:** 2025-05-20

**Authors:** Cheng Yan, Jianzhong Shi, Pengfei Cui, Yuan Chen, Congcong Wang, Yan Wang, Jiahao Miao, Yaping Zhang, Huihui Kong, Xianying Zeng, Guobin Tian, Chengjun Li, Yasuo Suzuki, Guohua Deng, Hualan Chen

**Affiliations:** a State Key Laboratory for Animal Disease Control and Prevention, Harbin Veterinary Research Institute, CAAS, Harbin, People’s Republic of China; b Institute of Western Agriculture, CAAS, Changji, People’s Republic of China; c National Poultry Laboratory Animal Resource Center, Harbin Veterinary Research Institute, CAAS, Harbin, People’s Republic of China; d Department of Medical Biochemistry, University of Shizuoka School of Pharmaceutical Sciences, Shizuoka, Japan

**Keywords:** Avian influenza virus, H3N3, evolution, guinea pig, transmission

## Abstract

Avian influenza viruses continue to challenge poultry and human health; therefore, careful surveillance and evaluation of emerging viruses are important for animal disease control and human influenza pandemic preparedness. In this study, we detected a series of H3N3 subtype avian influenza viruses in chickens, pigeons, and ducks during our routine surveillance and diagnosis between September 2022 and May 2023. We performed extensive analyses to fully understand the origins of these viruses and their risk to animals and humans. We found that the viruses were complex reassortants; the viruses from chickens and pigeons carry genes mainly derived from H3N8 viruses and H10N3 viruses, whereas the two duck viruses were reassortants of duck and wild bird viruses. The chicken and pigeon, but not duck, viruses replicated in multiple organs of chickens and were shed for up to 13 days, but none caused disease or death. Six of the viruses tested all bound to both avian- and human-type receptors. Seventeen viruses were tested in mice and most replicated efficiently but were not lethal. Six viruses were tested in guinea pigs, and four of them transmitted efficiently via respiratory droplets. Our study thus identified novel H3N3 avian influenza viruses and revealed their zoonotic potential, thereby emphasizing the importance of careful monitoring and control of H3 viruses in animals.

## Introduction

Influenza A virus, a single-stranded, segmented, negative-sense RNA virus, is classified into different subtypes based on the antigenicity of its viral glycoproteins: hemagglutinin (HA) and neuraminidase (NA). To date, 17 HA subtypes (H1–H16 and H19) and nine NA subtypes (N1–N9) have been identified from avian species [1,2], and subtypes H17N10 and H18N11 have been detected in bats [3,4]. Some strains of the H5 and H7 subtypes are highly pathogenic for poultry and have caused severe outbreaks in domestic poultry and wild birds [5–12]. H5 and H7 viruses also pose substantial threats to public health and have caused more than 2,600 documented human infections around the world [13].

Low pathogenic avian influenza viruses can also cause infection in mammals and pose a threat to human health [14]. In 2011, Ghazi Kayali et al. provided serological evidence of infection with H4 viruses among Lebanese chicken growers [15]. In 2013, H6N1 virus infection was detected in a 20-year-old woman in Taiwan, China [16]. In 2022–2023, the H3N8 viruses caused three human infections in China [17–19], and animal studies have shown that certain H3N8 strains can efficiently transmit in guinea pigs and ferrets via both direct contact and respiratory droplets [20,21]. H10 viruses carrying different NA subtypes have infected at least 11 people in Australia and China, and three people have died from the infection [22–25]. H9N2 viruses have caused more than 120 human infections across Bangladesh, Cambodia, China, Egypt, Ghana, India, Oman, Senegal, and Vietnam [26–29]. Studies have shown that naturally isolated H9N2 avian influenza viruses exclusively bind human-type receptors and are transmissible via respiratory droplets in different animal models [30–32]. The H9N2 virus “donates” its internal genes to other influenza virus subtypes, including H7N9, H3N8, H10N3, H10N8, and H5N6, making them more likely to infect humans [20,24,33,34]. Since the low pathogenic avian influenza viruses usually cause mild or asymptomatic infections in animal hosts, their eradication is not a priority for animal disease control in many countries, allowing them to evolve silently in nature and threaten human health.

Different H3 subtype influenza viruses widely circulate in nature. The H3N2 viruses have been a major cause of human influenza epidemics since 1968 [35,36]. They are also commonly found in pigs, dogs, wild birds, and domestic poultry. H3N8 virus is one of the major pathogens circulating in horses and donkeys [37,38]. In the past few years, H3N8 viruses that are different from the mammalian lineage were detected in poultry in China and caused three human infections [17–19]. During our routine surveillance between September 2022 and May 2023, we found that the H3N8 viruses had disappeared, but H3N3 viruses were detected in several provinces. In this study, we investigated how these viruses emerged and examined their genetics, receptor-binding properties, replication and transmissibility in different animals.

## Materials and methods

### Ethics statements

The protocols for animal studies were approved by the Animal Experiments Ethics Committee of the Harbin Veterinary Research Institute of the Chinese Academy of Agricultural Sciences (HVRI, CAAS).

### Virus isolation

The samples collected during our routine surveillance were processed at the enhanced level 2 biosafety facility (BSL2 +) at HVRI, CAAS. Oropharyngeal and cloacal swabs of the same bird were placed in the same sample collection tube and counted as one swab sample. Swab samples, fecal samples, or organs of diseased or dead birds were individually inoculated into 10-day-old embryonated chicken eggs and incubated for 48 h at 37℃. The HA subtype was identified by using the hemagglutination inhibition (HI) test and the NA subtype was confirmed by direct sequence analysis. Viral stocks were grown in specific-pathogen-free (SPF) chicken eggs and kept at −70℃.

### Genetic and phylogenetic analyses

The genome of the H3N3 viruses was sequenced on an Applied Biosystems DNA analyzer (3500xL Genetic analyzer, United States of America). The nucleotide sequences were edited with the SeqMan module of the DNAStar package. The time-scaled phylogenetic tree of the HA gene was inferred using an asymmetric continuous-time Markov chain with Bayesian stochastic search variable selection implemented in BEAST (v1.10.4). The maximum likelihood tree of HA gene sequences and the phylogenetic analysis of NA and the six internal genes was performed using IQ-TREE with the fast bootstrap method (the ultrafast bootstrap value set to 10,000); 95% sequence identity cut-off points were used to categorize gene groups in phylogenetic trees.

### Receptor-binding assay

The receptor-binding specificity of the H3N3 viruses was analyzed using a solid-phase direct binding assay as previously described [39–41]. The two different glycopolymers, α-2,3-sialylglycopolymer (avian-type receptor) and α-2,6-sialylglycopolymer (human-type receptor), used in this study were synthesized by Yamasa Corporation Co. Ltd., Japan. Chicken antisera against the A/chicken/Guangdong/S1286/2009 (H3N8) virus, A/chicken/Chongqing/SD001/2021 (H5N6) virus, and A/swine/Jiangxi/261/2016 (H1N1) virus were used as primary antibodies, and horseradish peroxidase (HRP)-conjugated goat-anti-chicken antibody (Sigma-Aldrich, St. Louis, MO, USA) was used as the secondary antibody.

### Chicken study

Six-week-old SPF White Leghorn chickens (National Poultry Laboratory Animal Resource Center, Harbin, China) and 22-week-old commercial Hy-Line Brown laying hens that were serologically negative for influenza viruses were used to determine the replication and virulence of the H3N3 viruses. Groups of 13 chickens housed in isolated cages were inoculated intranasally (i.n.) with 10^6^ 50% egg infective dose (EID_50_) of each H3N3 virus in a volume of 0.1 mL. On day 3 post-inoculation (p.i.), three chickens from each group were killed and their organs (larynx, trachea, lung, heart, livers, spleen, kidneys, pancreas, bursa, cecum, brain, thymus, or oviduct) were harvested for virus titration in chicken eggs. The remaining 10 chickens were monitored for signs of disease and death for 14 days. Oropharyngeal and cloacal swabs of these chickens were collected on days 3, 5, 7, 9, 11, and 13 p.i. to assess virus shedding. The viral titres were calculated using the method of Reed and Muench. The sera of the surviving chickens were collected for H3 antibody detection.

### Mouse study

Groups of eight 6-week-old BALB/c female mice (Beijing Experimental Animal Center, Beijing, China) were lightly anesthetized with CO_2_ and inoculated i.n. with 10^6^ EID_50_ of H3N3 viruses in a volume of 50 μL. On day 3 p.i., three mice in each group were euthanized and their organs (nasal turbinate, lung, spleen, kidneys, and brain) were collected for virus titration in chicken eggs. The remaining five mice in each group were monitored daily for body weight loss and survival for two weeks. The sera of the surviving mice were collected for H3 antibody detection.

### Replication and transmission in guinea pigs

Hartley strain female guinea pigs weighing 300–350 g (Beijing Experimental Animal Center, Beijing, China) and serologically negative for influenza viruses were used in this study. Groups of two guinea pigs anesthetized with Ketamine (20 mg/kg) and xylazine (1 mg/kg) were inoculated i.n. with 10^6^ EID_50_ of each test virus in a volume of 300 μL (150 μL per nostril). The animals were euthanized on day 3 p.i. and their nasal washes, trachea, and lung tissues were collected for virus titration in chicken eggs.

The respiratory droplet transmission study was carried out with three pairs of “1:1” guinea pigs as described previously [42]. Briefly, groups of three guinea pigs were inoculated i.n. with 10^6^ EID_50_ of virus in a 300 μL volume (150 μL per nostril) and housed in three separate cages within an isolator; 24 h later, three naive guinea pigs in each group were placed in three adjacent cages (4-cm away). Nasal washes were collected every two days p.i. to detect virus replication, and sera were collected from all animals on day 21 p.i. to assess seroconversion.

## Results

### Isolation and identification of H3N3 viruses

Between September 2022 and May 2023, during our routine surveillance and diagnosis, we isolated 975 strains of avian influenza viruses from 39,862 swab samples and 192 organ samples of diseased or dead birds collected from live poultry markets, poultry farms, and poultry slaughterhouses in 27 provinces. Fifty-six strains were confirmed as H3N3 viruses, including 49 strains isolated from apparently healthy poultry at live poultry markets, one strain isolated from a domestic duck on a farm, and six strains isolated from the lung samples of sick or dead chickens that were submitted to our laboratory for diagnostic purposes.

### Phylogenetic analysis of the HA gene of H3 avian influenza viruses

To understand how the H3N3 viruses emerged and reveal their genetic relationships, we selected 24 representative viruses isolated from different sampling times, locations, and host species (Table 1) and sequenced their genomes [sequence data of these viruses have been deposited in the databases of the Global Initiative on Sharing All Influenza Data (GISAID; https://www.gisaid.org); accession numbers are EPI4314271-EPI4314462].

**Table 1.** Sample information and genotypes of the H3N3 viruses sequenced in this study.

Sample information					Virus		
Collected date	Location	Source	Type	Avian species	Full name	Abbreviation	Genotype
October 26, 2022	Poultry market	Surveillance	Swab	Layer	A/chicken/Jiangsu/S4625/2022	CK/JS/S4625/22	G1
October 26, 2022	Poultry market	Surveillance	Swab	Layer	A/chicken/Jiangsu/S4566/2022	CK/JS/S4566/22	G2
October 26, 2022	Poultry market	Surveillance	Swab	Layer	A/chicken/Jiangsu/S4616/2022	CK/JS/S4616/22	G2
November 1, 2022	Poultry market	Surveillance	Swab	Broiler	A/chicken/Jiangsu/S4651/2022	CK/JS/S4651/22	G2
November 1, 2022	Poultry market	Surveillance	Swab	Duck	A/duck/Jiangsu/S4716/2022	DK/JS/S4716/22	G2
November 9, 2022	Poultry market	Surveillance	Swab	Layer	A/chicken/Zhejiang/S4716/2022	CK/ZJ/S4716/22	G1
November 9, 2022	Poultry market	Surveillance	Swab	Duck	A/duck/Jiangxi/S4312/2022	DK/JX/S4312/22	G10
February 16, 2023	Poultry market	Surveillance	Swab	Layer	A/chicken/Zhejiang/S1353/2023	CK/ZJ/S1353/23	G1
February 16, 2023	Poultry market	Surveillance	Swab	Goose	A/goose/Zhejiang/S1362/2023	GS/ZJ/S1362/23	G1
February 16, 2023	Poultry market	Surveillance	Swab	Duck	A/duck/Zhejiang/S1437/2023	DK/ZJ/S1437/23	G5
February 20, 2023	Poultry market	Surveillance	Swab	Duck	A/duck/Yunnan/S1312/2023	DK/YN/S1312/23	G1
February 20, 2023	Poultry market	Surveillance	Swab	Broiler	A/chicken/Yunnan/S1328/2023	CK/YN/S1328/23	G1
February 20, 2023	Poultry market	Surveillance	Swab	Broiler	A/chicken/Yunnan/S1369/2023	CK/YN/S1369/23	G4
February 20, 2023	Poultry market	Surveillance	Swab	Broiler	A/chicken/Yunnan/S1385/2023	CK/YN/S1385/23	G3
February 28, 2023	Poultry market	Surveillance	Swab	Layer	A/chicken/Fujian/S1106/2023	CK/FJ/S1106/23	G8
February 28, 2023	Poultry market	Surveillance	Swab	Pigeon	A/pigeon/Fujian/S1248/2023	PG/FJ/S1248/23	G8
February 28, 2023	Farm	Surveillance	Swab	Duck	A/duck/Hunan/S1488/2023	DK/HuN/S1488/23	G9
March 3, 2023	Farm	Diagnosis	Lung	Layer	A/chicken/Jiangsu/SD13/2023^a^	CK/JS/SD13/23	G1
March 15, 2023	Poultry market	Surveillance	Swab	Layer	A/chicken/Jiangsu/S1402/2023	CK/JS/S1402/23	G2
March 15, 2023	Poultry market	Surveillance	Swab	Pigeon	A/pigeon/Jiangxi/S1621/2023	PG/JX/S1621/23	G7
March 15, 2023	Poultry market	Surveillance	Swab	Layer	A/chicken/Jiangxi/S1670/2023	CK/JX/S1670/23	G6
March 16, 2023	Poultry market	Surveillance	Swab	Broiler	A/chicken/Jiangsu/S1741/2023	CK/JS/S1741/23	G1
April 22, 2023	Farm	Diagnosis	Lung	Layer	A/chicken/Heilongjiang/SD37/2023^a^	CK/HLJ/SD37/23	G1
May 31, 2023	Farm	Diagnosis	Lung	Layer	A/chicken/Hebei/SD59/2023^a^	CK/HeB/SD59/23	G1

^a^ The virus was isolated from the lungs of a sick or dead chicken submitted to our laboratory for diagnosis.

We constructed the maximum likelihood phylogenetic tree of the H3 avian influenza virus HA gene to study the evolution of the HA gene of the H3 subtype avian influenza virus globally over the past decade. The HAs of 534 H3 viruses detected across 21 countries were used, including the 24 H3N3 viruses sequenced in this study and 510 H3 avian influenza representative strains downloaded from the GISAID EpiFlu database, which include the three H3N8 viruses that infected humans in China [17–19].

**Figure 1.**
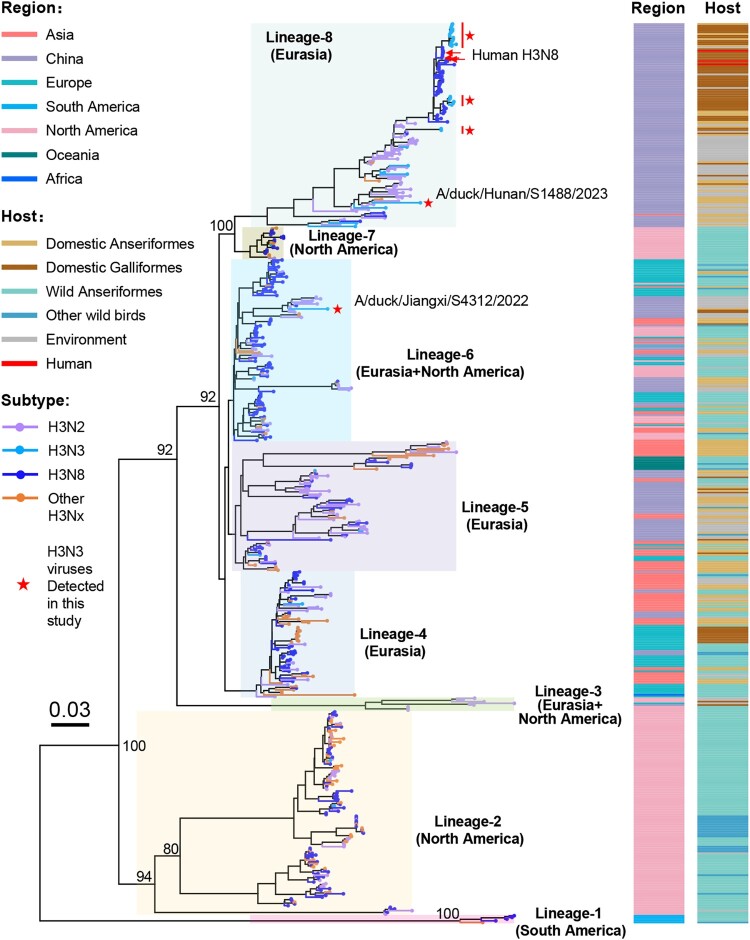
Phylogenetic analysis of the HA gene of H3 avian influenza viruses. Maximum-likelihood phylogenetic tree of the HA genes of 534 H3 viruses, including those of 24 H3N3 viruses sequenced in this study, three human H3N8 viruses, and 507 H3 viruses downloaded from the GISAID EpiFlu database. Domestic Anseriformes: duck and goose. Domestic Galliformes: chicken, turkey, gallus, and pigeon. Wild Anseriformes: American wigeon, European wigeon, blue-winged teal, cinnamon teal, common teal, green-winged teal, gray teal, Baikal teal, American black duck, mallard duck, gadwall, garganey, bufflehead, long-tailed duck, Muscovy duck, shoveler, pink-eared duck, ruddy duck, tufted duck, yellow-billed pintail, northern pintail, barnacle goose, bean goose, graylag goose, Egyptian goose, and emperor goose.

We found that the HA genes of these H3 viruses formed eight lineages (Figure 1, Figure S1). The viruses carrying the HA gene of lineage-1 were all detected in South America, the viruses carrying the HA gene of lineage-2 and lineage-7 were all detected in North America, the viruses carrying the HA gene of lineage-4, -5, and -8 were all detected in Eurasian countries. However, the viruses carrying the HA gene of lineage-3 and lineage-6 were detected in migratory birds and domestic birds in both Eurasia and North America, indicating that, similar to the highly pathogenic H5 viruses [43,44], H3 avian influenza viruses are also frequently transmitted between Eurasia and North America. Of note, the HA genes of 23 of the H3N3 viruses detected in this study belong to lineage-8, whereas the HA of the A/duck/Jiangxi/S4312/2022 virus belongs to lineage-6 (Figure 1, Figure S1).

### Genotypic analysis of the H3N3 viruses

**Figure 2.**
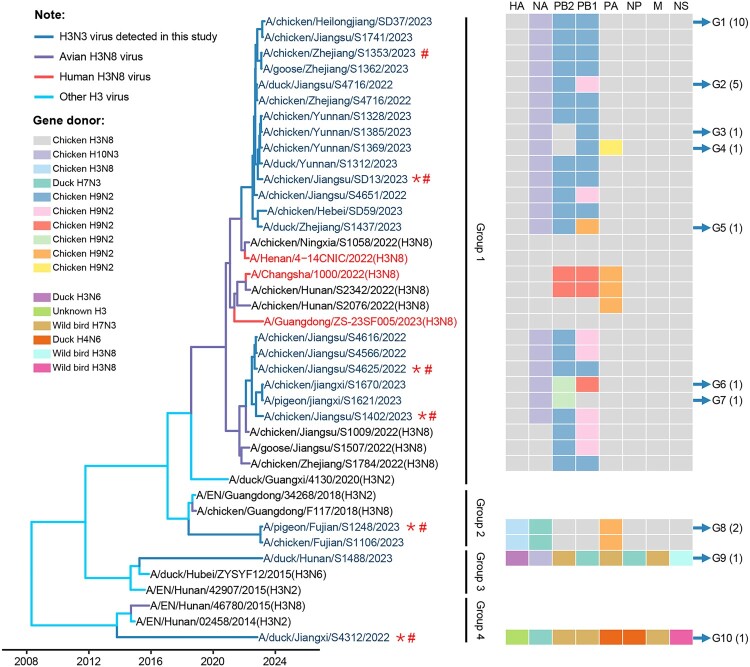
Genotypic analysis of the H3N3 avian influenza viruses. The time-scaled phylogenetic tree of the HA genes was inferred with 40 viruses, including 24 H3N3 viruses detected in this study, three human H3N8 viruses, six avian H3N8 viruses, and seven representative viruses downloaded from the GISAID EpiFlu Database. * Indicates the virus was tested in chickens; **^#^** indicates the virus was tested in guinea pigs.

The HA genes of the 24 H3N3 viruses in this study shared 85.5%–100% identity at the nucleotide level and formed four distinct phylogenetic groups (Figure 2). The neuraminidase (NA), basic polymerase 2 (PB2), basic polymerase 1 (PB1), acidic polymerase (PA), nucleoprotein (NP), matrix (M), and nonstructural protein (NS) genes of the 24 H3N3 viruses shared 91%–100%, 85%–100%, 87.3%–100%, 89.7%–100%, 86.1%–100%, 89.5%–100%, and 69.5%–100% identity, respectively, and formed 2–7 groups in their phylogenetic trees (Figure S2). Based on their phylogenetic diversity, the 24 H3N3 viruses were classified into ten genotypes (G1 to G10) (Figure 2, Table 1).

As shown in Figure 2, the 20 viruses carrying the group-1 HA genes have similar NA, NP, M, and NS genes. These 20 viruses derived their genes from the H3N8, H10N3, and H9N2 viruses that were previously detected in chickens in China [21,24,45,46] and formed seven different genotypes (G1–G7; Figure 2, Figure S2). The two viruses carrying the group-2 HA genes (G8) are reassortants that derived their genes from the H3N8 chicken viruses, H7N3 duck viruses, and H9N2 viruses that were previously detected in China [21,46,47] (Figure 2, Figure S2). The two H3N3 duck viruses derived all their genes from duck and wild bird viruses (Figure 2; Figure S2) and formed two different genotypes (G9 and G10). These results show that the H3N3 viruses detected in poultry included reassortant viruses of H3N8 and H9N2 chicken viruses previously detected in poultry, as well as new viruses introduced into ducks from wild birds.

### Replication and virulence of the H3N3 viruses in chickens

**Figure 3.**
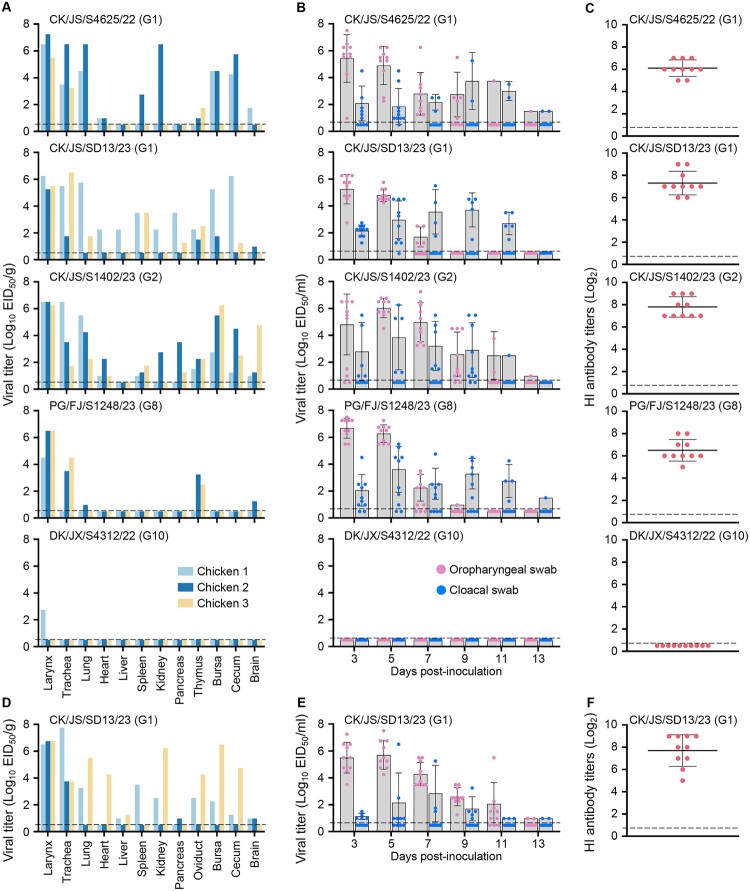
Replication and virulence of the H3N3 viruses in chickens. (A) Viral titres in organs of SPF chickens. (B) Viral shedding in swabs from SPF chickens. (C) HI antibody titres of SPF chickens. (D) Viral titres in organs of layer chickens. (E) Viral shedding in swabs from layer chickens. (F) HI antibody titres in layer chickens.

Low pathogenic avian influenza viruses usually cause mild or asymptomatic infections in poultry; however, some of the H3N3 viruses in this study were isolated from the lungs of sick or dead chickens from farms. To determine whether the disease was caused by the H3N3 virus alone or a co-infection with other pathogens on the farm, we tested five representative viruses, including three chicken viruses, one pigeon virus, and one duck virus in SPF chickens. We found that these viruses have different replication patterns in chickens. The three chicken viruses replicated efficiently and were detected in multiple organs or tissues of chickens; the pigeon virus was detected in the respiratory tract, thymus, and brain, but not in any other organs tested; and the duck virus replicated very poorly in chickens and was detected only in the oropharynx of one chicken (Figure 3(a)).

Virus shedding and seroconversion were also evaluated in all virus-inoculated chickens. Virus shedding was detected in the oropharyngeal and cloacal swabs of the chickens inoculated with the three chicken viruses or the pigeon virus for up to 13 days post-inoculation but was not detected from any chickens that were infected with the duck virus (Figure 3(b)). All chickens remained apparently health and survived the duration of the two-week observation period. The chickens inoculated with the chicken viruses and pigeon virus all seroconverted, whereas none of the duck virus-inoculated chickens seroconverted (Figure 3(c)).

We also investigated whether the H3N3 viruses could cause disease in farmed chickens. A group of 13 22-week-old commercial Hy-Line brown laying hens were inoculated with the chicken virus CK/JS/SD13/23, which was isolated from a dead chicken. Virus replication and shedding in the farmed chickens were similar to those in SPF chickens (Figure 3(d,e)), and all chickens survived and seroconverted (Figure 3(f)). These studies show that H3N3 viruses have different replication capabilities in chickens, and even when the virus replicates in multiple chicken organs, it does not cause illness or death.

### Replication and pathogenicity of H3N3 viruses in mice

**Figure 4.**
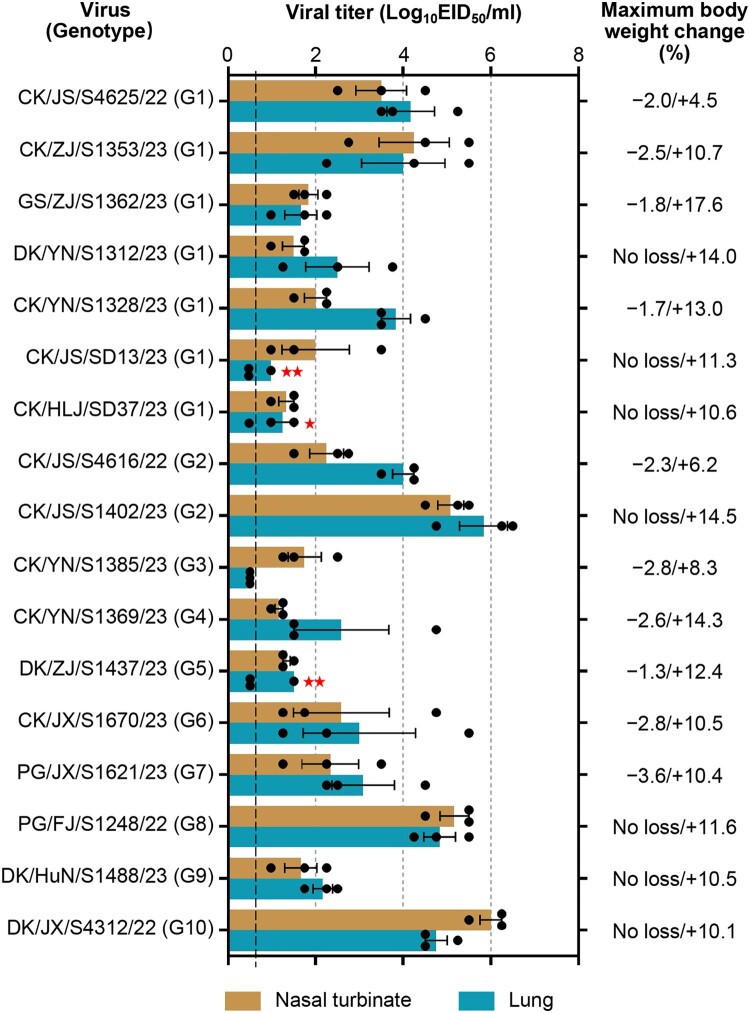
Replication and virulence of the H3N3 viruses in mice. Viral titres in organs of mice inoculated i.n. with 10^6^ EID_50_ of test viruses. Data shown are means ± standard deviations. The dashed line indicates the lower limit of detection. Maximum body weight changes were calculated as the difference between the initial values and the last values recorded for the infected mice during the two-week observation period.

Non-pathogenic avian influenza viruses can sometimes infect humans and cause serious problems [19,34,48]. To evaluate the potential risk posed by the emerging H3N3 viruses to humans, we investigate the replication and pathogenicity of 17 H3N3 strains in 6-week-old female BALB/c mice. As shown in Figure 4, 13 viruses replicated efficiently and were detected in the nasal turbinate and lungs of all mice that were euthanized on day 3 p.i. The other four strains were also detected in the nasal turbinate of all inoculated mice, but were not detected in the lungs or only were detected in the lungs of one or two of the three inoculated mice (Figure 4). The viral titres in the organs of the animals varied among the strains.

In addition, virus was not detected in the spleen, kidneys, or brain of mice inoculated with any of the viruses. Ten viruses infected mice lost 1.3%–3.6% body weight in the first week, but all mice survived and gained 4.5%–17.6% body weight at the end of the observation period. These results demonstrate that the H3N3 viruses can replicate in mice but are not fatal to them.

### Receptor-binding properties of the H3N3 viruses

**Figure 5.**
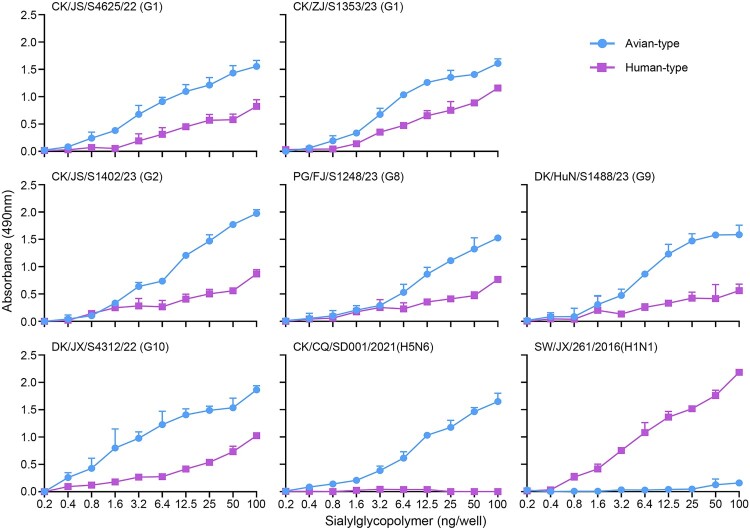
Receptor-binding properties of H3N3 viruses. The receptor-binding properties of H3N3 viruses were detected by using sialylglycopolymers (α2,3-sialylglycopolymer, blue; α2,6-sialylglycopolymer, purple). The data shown are the means of three repeats; the error bars indicate standard deviations. Two viruses, CK/CQ/SD001/2021 (H5N6) and SW/JX/261/2016 (H1N1), were used as controls.

Sialic acid receptor-binding preference is important for the replication and transmission of influenza virus, and binding to α2,6-linked sialic acids (SAs) is a prerequisite for an influenza virus to transmit efficiently among humans [49–51]. To investigate the receptor-binding properties of the H3N3 viruses, we tested the affinity of six representative H3N3 viruses that were isolated at different sampling times and locations, and from different hosts to two different glycopolymers using solid-phase binding assays as previously described [39]. We also included an H5N6 virus (CK/CQ/SD001/2021 (H5N6)) and an H1N1 virus (SW/JX/261/2016 (H1N1)) as controls. As shown in Figure 5, the H5N6 virus exclusively bound to the α2,3-sialglycopolymer (avian-type receptor), the H1N1 virus exclusively bound to the α2,6-sialglycopolymer (human-type receptor), whereas all six H3N3 viruses bound to both the avian- and human-type receptors, although their affinity for the avian-type receptor was greater than that for the human-type receptor (Figure 5).

### Replication and transmission of the H3N3 viruses in guinea pigs

**Figure 6.**
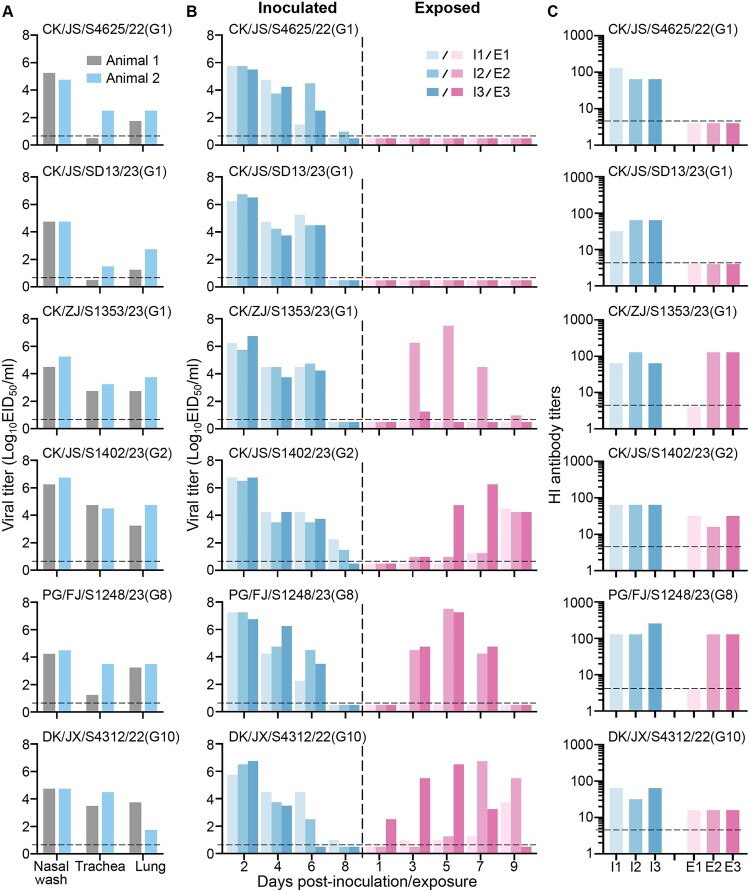
Replication and respiratory droplet transmission of H3N3 viruses in guinea pigs. (A) Replication of the H3N3 virus in the nasal turbinates, trachea, and lungs of guinea pigs. (B) Respiratory droplet transmission of H3N3 viruses in guinea pigs. (C) HI antibody titres of guinea pigs inoculated or exposed to the H3N3 virus. Each colour bar represents the viral titre from an individual animal. The horizontal dashed black lines indicate the lower limit of detection.

To investigate the transmissibility of the H3N3 viruses, we selected six viruses that replicate efficiently in mice and tested in guinea pigs, a commonly used animal model for assessing the transmission potential of influenza viruses in humans. We found that all six viruses replicated in the upper respiratory tract and lungs of guinea pigs, with viral titres ranging from 4.3 to 6.8 log_10_ EID_50_ in nasal washes, and with viral titres ranging from 0.5 to 4.8 log_10_ EID_50_ and 1.3 to 4.8 log_10_ EID_50_ in the trachea and lungs, respectively (Figure 6(a)). In the transmission study, the virus was detected for 6 to 8 days in the nasal washes of animals that were inoculated with any of the six viruses (Figure 6(b)); however, in the exposed animals, virus detection differed among the different groups. Virus was not detected in the nasal washes of any animals that had been exposed to CK/JS/S4625/22- or CK/JS/SD13/23-inoculated animals, but was detected in the nasal washes of two animals that had been exposed to CK/ZJ/S1353/23- or PG/FJ/S1248/23-inoculated animals, and in the nasal washes of all three animals that were exposed to CK/JS/S1402/23- or DK/JX/S4312/22-inoculated animals (Figure 6(b)). Seroconversion occurred in all virus-inoculated animals and the exposed animals that shed virus (Figure 6(c)). These results indicate that multiple H3N3 strains circulating in poultry can transmit among guinea pigs via respiratory droplets.

## Discussion

Gene reassortment between different strains of influenza virus is one of the most important mechanisms for virus evolution, and specific gene constellations could confer harmful biological characteristics on new viruses. Previous studies have shown that an H5N8 clade 2.3.4.4b virus reassorted with different low pathogenic viruses and generated an H5N1 clade 2.3.4.4b virus in wild birds in Europe [44,52], which subsequently spread to different continents and formed numerous genotypes of H5N1 viruses. Of the many genotypes produced in North America, the so-called genotype B3.13 virus jumped and quickly adapted to cows [53,54]. Studies also indicate that the internal genes of the H9N2 viruses made different subtypes of avian influenza viruses, including H7N9, H3N8, H10N3, H10N8, and H5N6, to easily infect humans [20,24,33,34]. The H3N3 viruses detected in this study are complex reassortants bearing genes from strains circulating in both domestic poultry and wild birds, and their receptor-binding and transmission properties suggest that, like H7N9 and H3N8 viruses, these emerging viruses pose a risk to humans.

Cui et al. tested the replication of different H3N8 viruses in chickens and detected some strains in the larynx and trachea of chickens, but none in the lungs or other organs [21]. In the present study, we found that some H3N3 viruses replicate efficiently in chickens and could be detected in multiple chicken organs (Figure 3). Genetic analyses indicated that these H3N3 viruses derived its HA gene from H3N8 virus, NA from H10N3 virus, and the other six gene segments (PB2, PB1, PA, NP, M, and NS) from previously detected H3N8 chicken viruses or H9N2 viruses (Figure 2). It remains to be seen whether the increased replication of H3N3 viruses in chickens is due to their gene constellation or due to substitutions in key amino acids. The DK/JX/S4312/22 is a new H3N3 reassortant of duck and wild bird viruses. DK/JX/S4312/22 barely replicates in chickens, making it a possible model virus for exploration of the genetic basis for chicken adaptation of duck and wild bird viruses. Moreover, DK/JX/S4312/22 replicates efficiently in mice and transmits in guinea pigs, suggesting that it may not need to “acquire” genes from the H9N2 virus to infect humans directly.

Avian influenza virus could easily acquire key substitutions in its PB2 to facilitate its adaptation to mammalian hosts [55–57]. Shi et al. reported that over 80% of the H7N9 avian influenza viruses isolated from humans obtained the E627K or D701N substitutions in their PB2 [11], which dramatically increased the replication of the virus in the lungs of the infected humans and caused severe disease or death [58]. Liang et al. demonstrated that the emergence of the PB2 E627K substitution in H7N9 virus is driven by the intrinsic low polymerase activity conferred by the viral PA protein [59]. When we sequenced the PB2 of the H3N3 viruses that we recovered from the lungs of mice, we also detected the E627K, E627V, or D701N substitution in some samples (Table S1), implying that if these viruses were to jump to humans, they could acquire similar substitutions and become more harmful.

Although the H3N3 viruses did not cause disease in chickens in our laboratory tests, their pathogenicity may increase when co-infected with other pathogens on chicken farms. Given the risks posed to the poultry industry and human health, in addition to careful monitoring, appropriate strategies to control emerging H3N3 viruses should be developed and implemented.

## Supplementary Material

Yan Fig S2dR1.tif

Yan Fig S2fR1.tif

Yan Fig S1R1.pdf

Yan Table S1.docx

Yan Fig S2gR1.tif

Yan Fig S2eR1.tif

Yan Fig S2aR1.tif

Yan Fig S2bR1.tif

Yan Fig S1R1.tif

Yan Fig S2cR1.tif
