# Supplementary figures and images for: Characterization of emerging H3N3 avian influenza viruses in poultry in China
Source: Emerg Microbes Infect. 2025 May 20;14(1):2509748. doi: 10.1080/22221751.2025.2509748 (PMC12128136; doi:10.1080/22221751.2025.2509748)

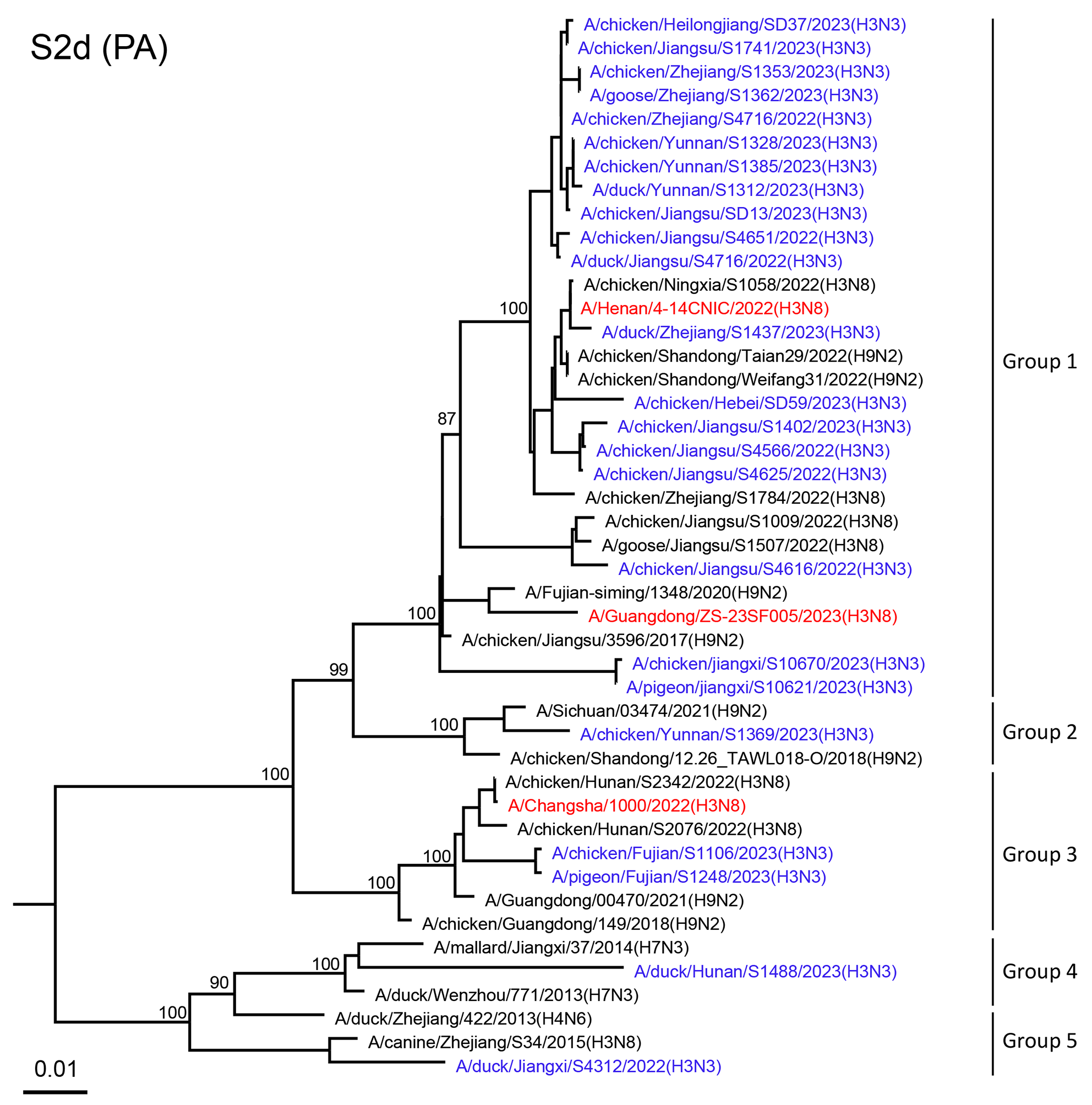

Supplement: Yan Fig S2dR1.tif [file TEMI_A_2509748_SM5700.tif]

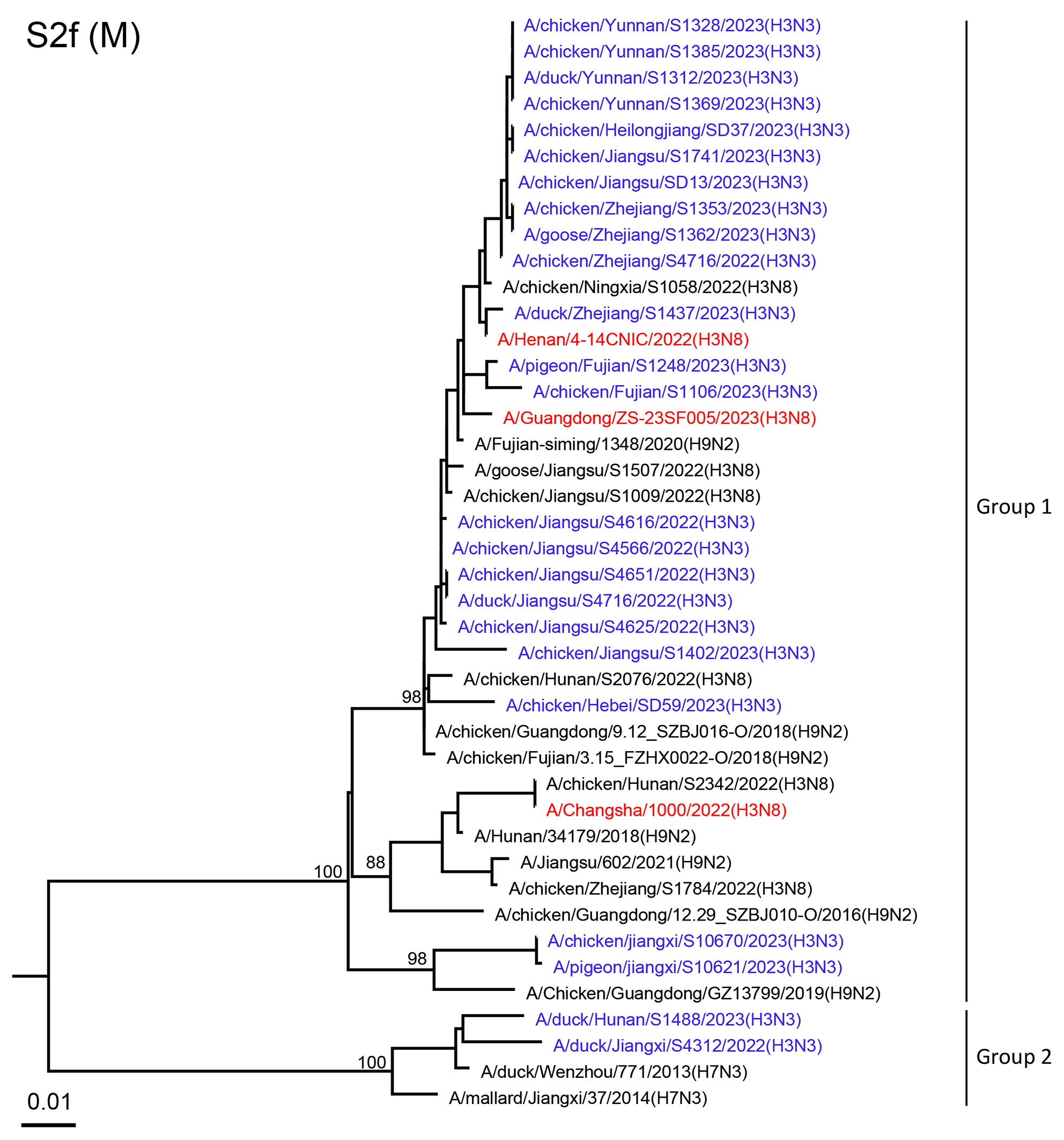

Supplement: Yan Fig S2fR1.tif [file TEMI_A_2509748_SM5699.tif]

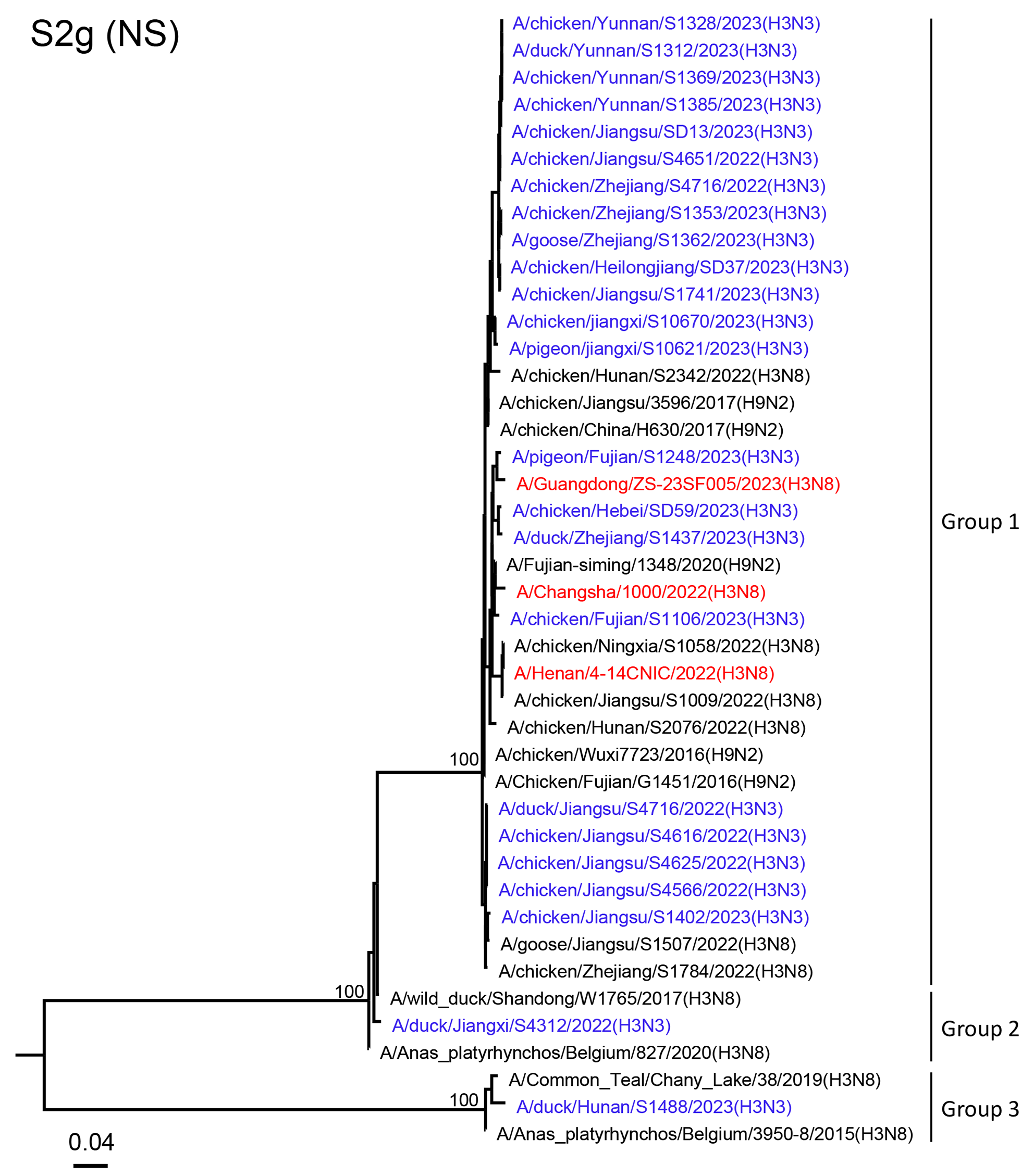

Supplement: Yan Fig S2gR1.tif [file TEMI_A_2509748_SM5696.tif]

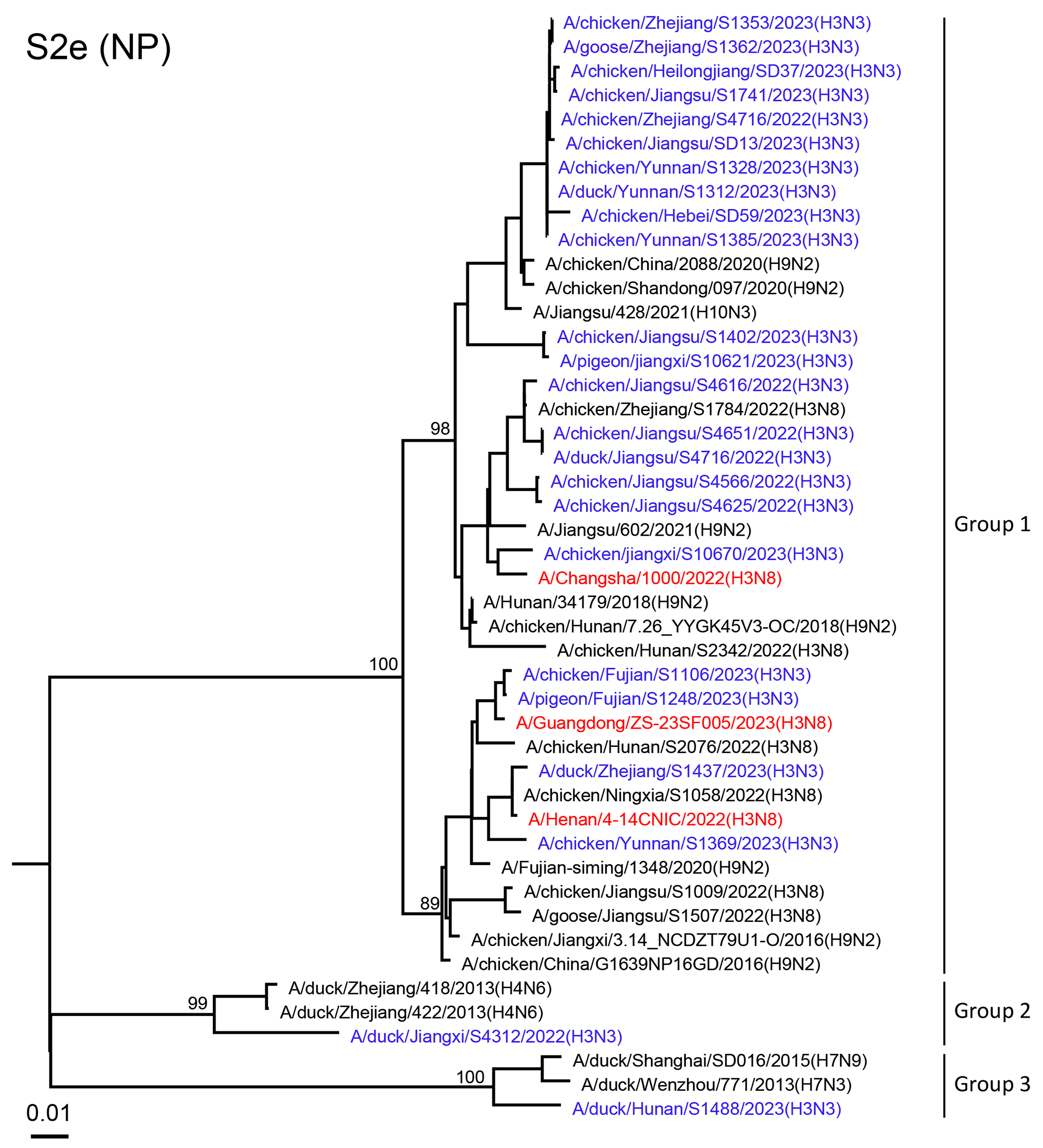

Supplement: Yan Fig S2eR1.tif [file TEMI_A_2509748_SM5695.tif]

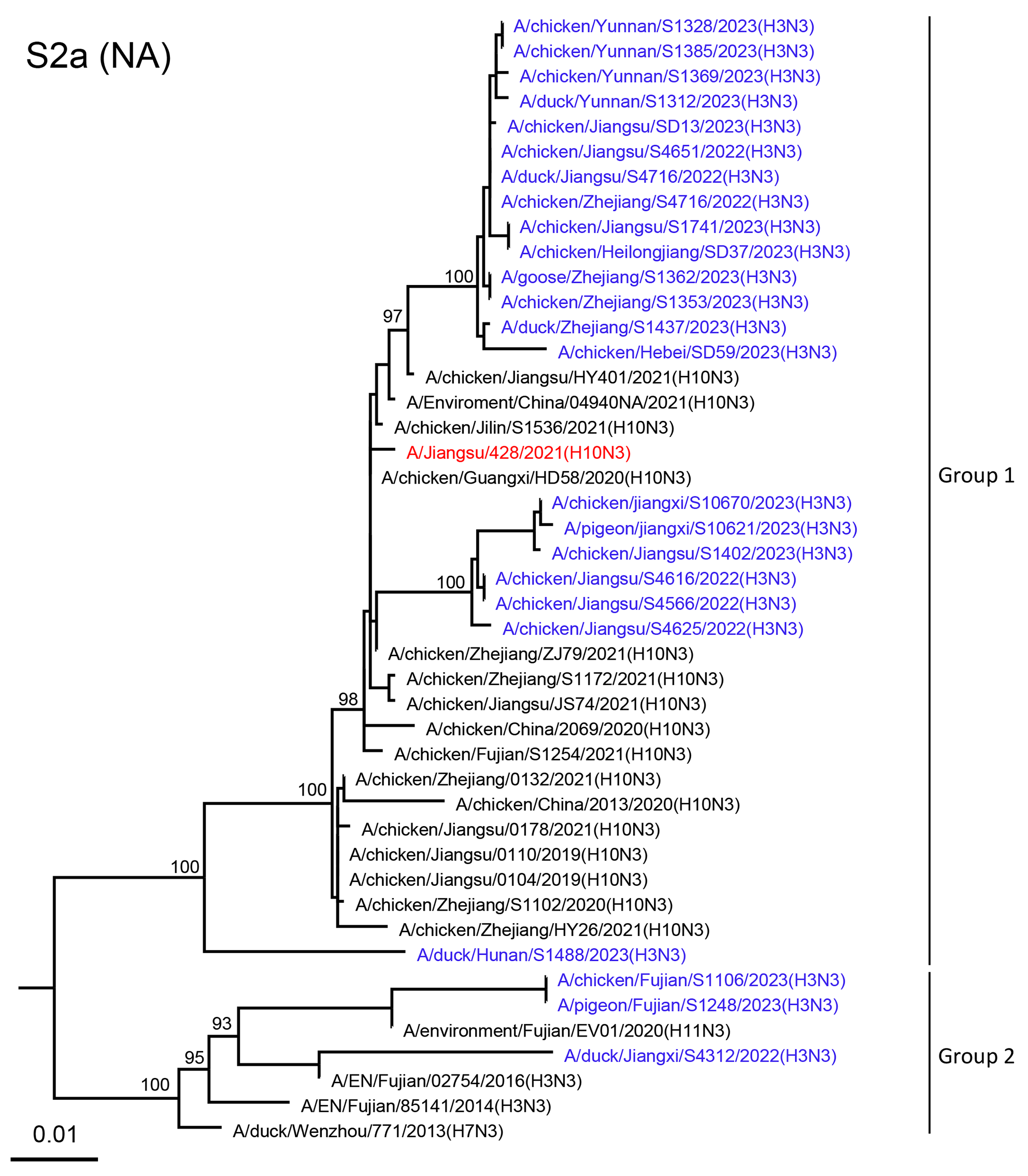

Supplement: Yan Fig S2aR1.tif [file TEMI_A_2509748_SM5694.tif]

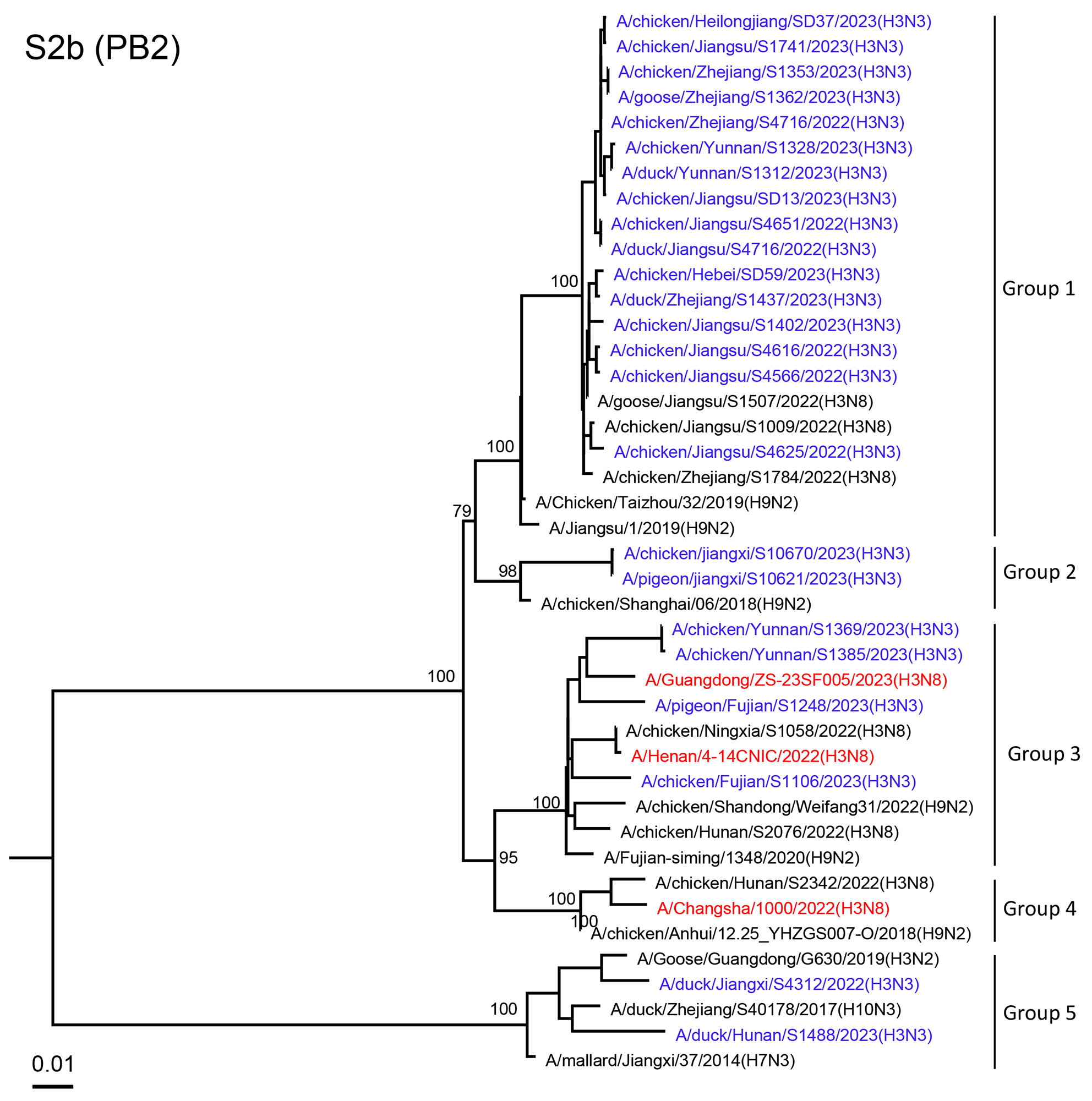

Supplement: Yan Fig S2bR1.tif [file TEMI_A_2509748_SM5693.tif]

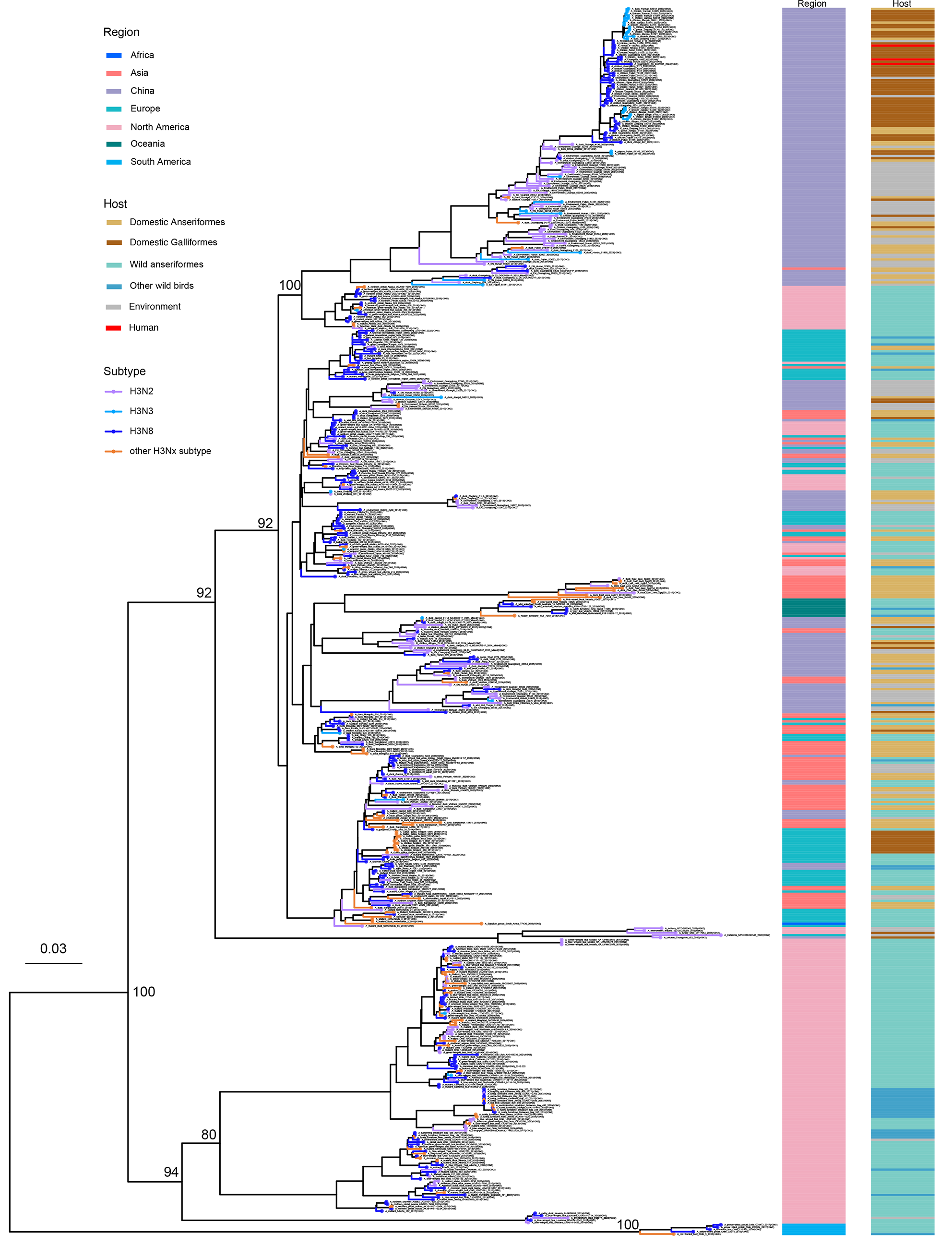

Supplement: Yan Fig S1R1.tif [file TEMI_A_2509748_SM5692.tif]

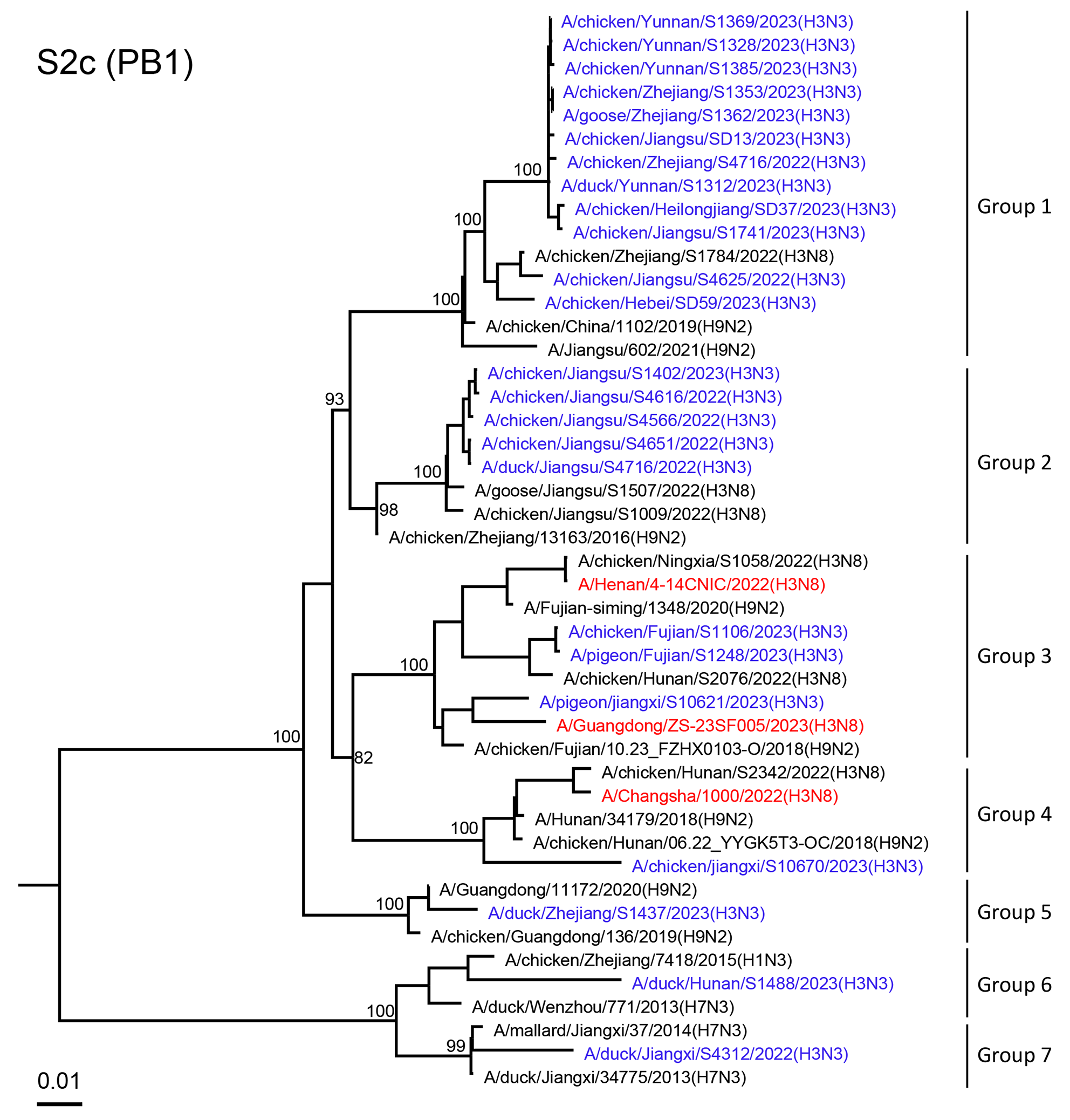

Supplement: Yan Fig S2cR1.tif [file TEMI_A_2509748_SM5691.tif]
