# Supplementary material for: Characterization of emerging H3N3 avian influenza viruses in poultry in China
Source: Emerg Microbes Infect. 2025 May 20;14(1):2509748. doi: 10.1080/22221751.2025.2509748 (PMC12128136; doi:10.1080/22221751.2025.2509748)
Supplement: Yan Fig S1R1.pdf [file TEMI_A_2509748_SM5698.pdf]

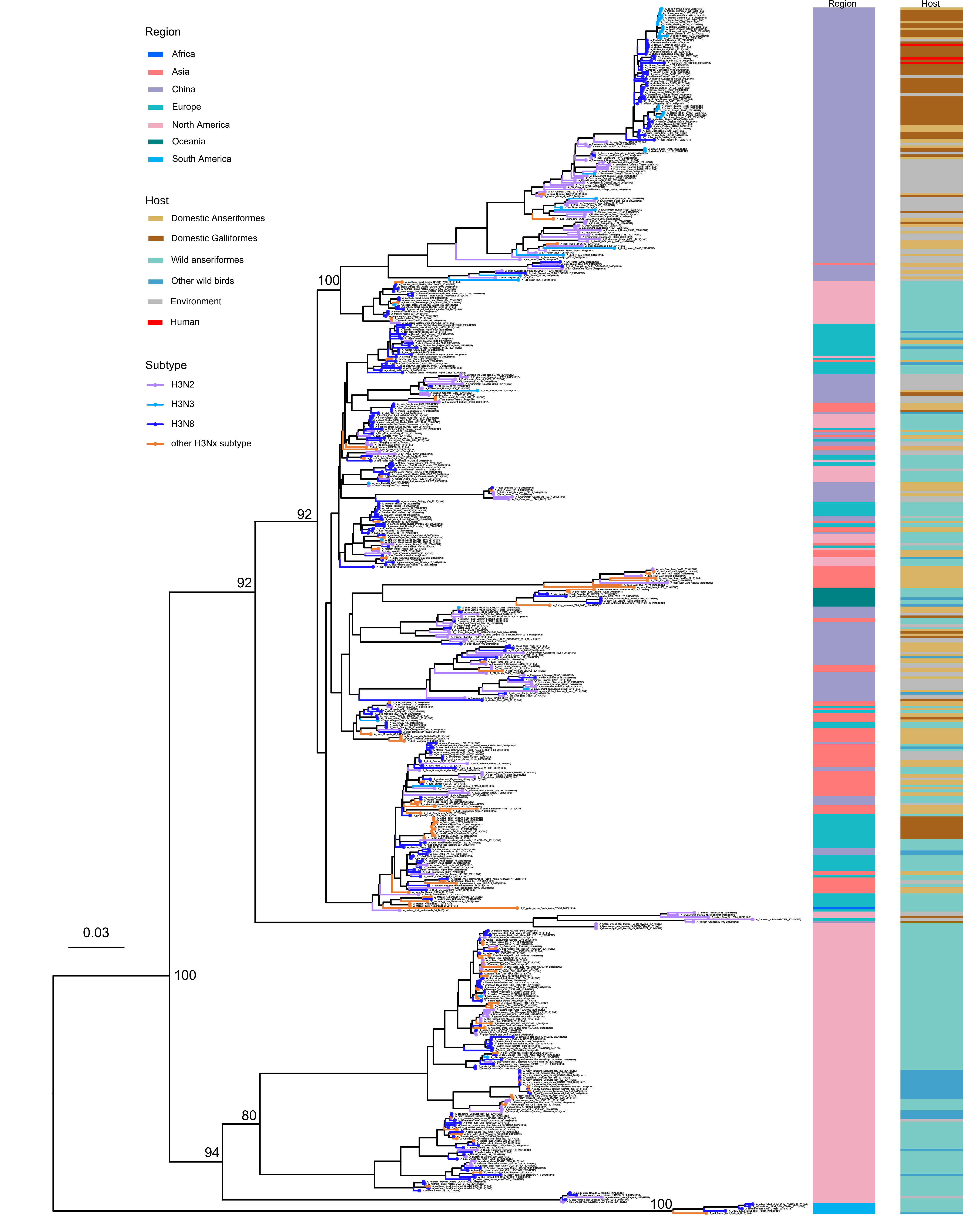
Region
Host
Region
Africa
Asia
China
Europe
North America
Oceania
South America
Host
Domestic Anseriformes
Domestic Galliformes
Wild anseriformes
Other wild birds
Environment
Human
Subtype
H3N2
H3N3
H3N8
other H3Nx subtype
100
92
92
100
80
94
100
0.03
