# Supplementary material for: Characterization of emerging H3N3 avian influenza viruses in poultry in China
Source: Emerg Microbes Infect. 2025 May 20;14(1):2509748. doi: 10.1080/22221751.2025.2509748 (PMC12128136; doi:10.1080/22221751.2025.2509748)
Supplement: Yan Table S1.docx [file TEMI_A_2509748_SM5697.docx]

Table S1. Amino acid substitutions in the PB2 of viruses recovered from the lungs of mice that were inoculated with H3N3 viruses.

| Virus | Mouse 1 | Mouse 2 | Mouse 3 |
| --- | --- | --- | --- |
| CK/JS/S4625/22 (G1) | 627K | - | - |
| CK/ZJ/S1353/23 (G1) | - | 701N | 627K |
| DK/YN/S1312/23 (G1) | - | - | 627K |
| CK/JS/S1402/23 (G2) | - | 627K | 627K |
| CK/YN/S1369/23 (G4) | - | - | 627K |
| CK/JX/S1670/23 (G6) | - | 627V | - |
| CK/JX/S1621/23 (G7) | - | - | 627K |

-, No substitution was detected.
